# Prognostic Significance of Lactate Clearance in Cardiogenic Pulmonary Edema in the Emergency Department

**DOI:** 10.3390/medicina60091502

**Published:** 2024-09-14

**Authors:** Mehmet Göktuğ Efgan, Ejder Saylav Bora, Ahmet Kayalı, Umut Payza, Tutku Duman Şahan, Zeynep Karakaya

**Affiliations:** Department of Emergency Medicine, Faculty of Medicine, Izmir Katip Çelebi University, 35100 Izmir, Turkey; saylavbora@hotmail.com (E.S.B.); ahmet.kayali@gmail.com (A.K.); umutpayza@hotmail.com (U.P.); tutkuduman75@gmail.com (T.D.Ş.); zeynepkarakaya76@hotmail.com (Z.K.)

**Keywords:** acute cardiogenic pulmonary edema, lactate clearance, emergency department

## Abstract

*Background and Objectives*: Acute cardiorespiratory failure disrupts the delicate balance of energy supply, demand, and consumption, with elevated lactate levels and decreased blood pH serving as crucial indicators. Acute cardiogenic pulmonary edema (ACPO), a common cause of acute respiratory failure, poses a substantial mortality risk. Lactate, a byproduct of pyruvate reduction, is a pertinent marker in perfusion assessment. Lactate clearance (LC) has proven prognostic efficacy in various conditions but lacks consensus on its predictive power in acute cardiogenic pulmonary edema. *Materials and Methods*: This prospective observational study, conducted in a metropolitan area’s third-level emergency department, involved patients with cardiogenic pulmonary edema from May 2021 to August 2023. The inclusion criteria specified acute cardiogenic pulmonary edema, excluding patients with incomplete data or other respiratory conditions. Lactate clearance, calculated at presentation and after 6 h, served as the primary outcome predictor. Our data analysis employed logistic regression, the ROC curve, and statistical tests. *Results:* The cohort of 106 patients revealed that a lactate clearance below 14.29% was significantly associated with mortality. While 51.6% of survivors were discharged, LC’s predictive success for discharge was inconclusive. Logistic regression underscored the significance of lactate clearance, with a one-unit increase yielding a 5.55-fold probability of survival. The AUC for LC was 0.759. *Conclusions*: This study pioneers the exploration of lactate clearance in patients with acute cardiogenic pulmonary edema. LC below 14.29% signifies a poor prognosis, emphasizing its potential as an early treatment initiation marker. While acknowledging this study’s limitations, we advocate for further multicenter research to refine the understanding of lactate clearance in this context.

## 1. Introduction

The occurrence of acute cardiorespiratory failure, regardless of its etiology, results in a disruption of the equilibrium between energy supply, demand, and consumption. The presence of elevated lactate levels in conjunction with a decreased blood pH is a valuable indicator for assessing the severity of this discrepancy [1].

Acute cardiogenic pulmonary edema (ACPO) is a prevalent etiology of acute respiratory failure, accounting for approximately 10% to 20% of acute cardiac failure syndromes and carrying the potential for mortality [2]. Acute cardiogenic pulmonary edema typically manifests as the abrupt onset of dyspnea while at rest, accompanied by reduced exercise tolerance, rapid breathing, elevated heart rate, and inadequate oxygen levels. Elevated levels of endogenous catecholamines and the development of hypertension as a result of stress are frequently observed in individuals with preserved left ventricular function. The presence of coughing is commonly observed in these instances. The primary objective in these individuals is to ensure the sufficient oxygenation of tissues to mitigate the risk of organ dysfunction and the development of numerous organ failures [3].

Lactate is formed because of the reduction of pyruvate by the enzyme lactate dehydrogenase [4,5]. In healthy people, the plasma lactate level is below two mmol/L [6]. Thanks to blood gas analyzers, lactate concentration is an easily measurable marker indicating the perfusion impairment of tissues independent of blood pressure; for instance, pathologies causing hypoxia or hypoperfusion cause a rapid increase in the plasma lactate levels [7]. In recent studies, lactate clearance (LC), calculated using the lactate value, has come to the forefront as a highly successful prognostic marker in sepsis, gastrointestinal bleeding, and pulmonary thromboembolism [8,9,10].

The prompt removal of lactate overproduction from the bloodstream can be achieved if the perfusion and function of the kidneys and liver are close to normal [11,12]. The acquisition of a solitary lactate measurement does not possess the ability to enhance clinical outcomes. However, therapeutic approaches that have the potential to reduce arterial lactate levels may exhibit a correlation with improved clinical outcomes. This strategy aims to effectively mitigate the condition of global tissue hypoxia. The lactate clearance (LC) concept is intended to incorporate this idea. Numerous studies have consistently demonstrated that individuals with rapid lung cancer exhibit superior survival rates in comparison to those affected by a slower progression of the disease [13,14,15,16]. Nevertheless, the ability of LC to accurately predict mortality has yet to be clearly established, and there is a lack of consensus between various studies regarding this matter.

To the best of our knowledge, there is still no study on the prognostic predictive power of lactate clearance in patients admitted to the emergency department with acute cardiogenic pulmonary edema. The present study was conducted to clarify this situation.

## 2. Materials and Methods

### 2.1. Study Design

This prospective observational study was conducted at a third-level emergency department in a metropolitan area with a population of 6 million people. It focused on patients with cardiogenic pulmonary edema who sought medical attention in the emergency department between May 2021 and August 2023. Before the commencement of this study, ethical approval was obtained from the local university ethics committee.

### 2.2. Patients and Setting

#### Inclusion and Exclusion Criteria

The inclusion criteria encompassed patients diagnosed with acute cardiogenic pulmonary edema, defined by the presence of pulmonary alveolar/interstitial congestion with at least two of the following criteria evident in the chest X-ray and/or thorax computed tomography [16]:

1. Severe respiratory distress, worsening respiratory distress, persistent severe breathlessness, or orthopnea;
2. Rales in the lungs;
3. High jugular venous pressure.

**Figure 1:**
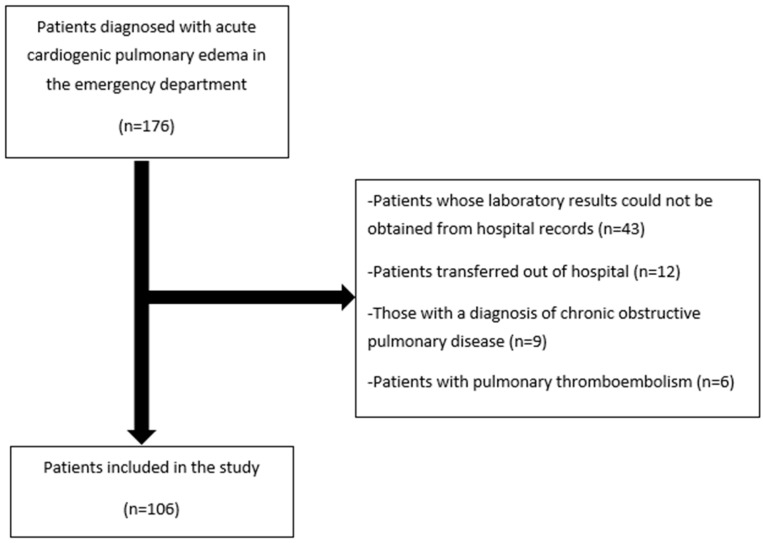
Consultation diagram.

Patient selection relied on reports of evaluations conducted by a cardiologist in the emergency department. The exclusion criteria involved patients with incomplete data, those referred to an external center, and individuals with chronic obstructive pulmonary disease or pulmonary thromboembolism, as illustrated in Figure 1.

### 2.3. Data Collection

Data collection involved patients meeting the study criteria, where vital signs, demographic information, and laboratory results of individuals diagnosed with acute cardiogenic pulmonary edema were meticulously recorded on dedicated patient forms. Additional information, including the diagnostic criteria from cardiological consultation notes, patients’ hospitalization status, intensive care requirements, and disease outcomes, was also documented.

### 2.4. Lactate Clearance Definition

Lactate clearance was assessed both at the time of presentation to the emergency department and at the 6 h mark. The lactate clearance calculation employed the formula [(Initial lactate − Final lactate)/Initial lactate] × 100 [17]. These data were subsequently analyzed to gauge their predictive power concerning the need for intensive care and mortality.

### 2.5. Outcomes

The primary outcome of this study focused on observing the predictive power of lactate clearance for mortality in patients initially presenting to the emergency service. The secondary outcome explored the potential of lactate clearance as a marker for hospital discharge. These outcomes were assessed to provide comprehensive insights into the prognostic value of lactate clearance in the context of acute cardiogenic pulmonary edema.

### 2.6. Statistical Analysis

Data were analyzed using IBM SPSS Statistics Standard Concurrent User V 26 (IBM Corp., Armonk, NY, USA) and MedCalc^®^ Statistical Software version 19.6 (MedCalc Software Ltd., Ostend, Belgium). Descriptive statistics were expressed as the number of units (n), percentage (%), mean ± standard deviation (mean ± sd), median (M), minimum (min), maximum (max), and interquartile range (IQR) values. 

The normal distribution of the numerical variables was evaluated by the Shapiro–Wilk normality test. In the comparison of control and thyroid groups, the independent sample *t* test was used for variables with a normal distribution, and the Mann–Whitney U test was used for variables without a normal distribution. Fisher’s exact test and the chi-square test were used to compare the groups with categorical variables.

A model was created with the variables’ effect on the outcome, and this model was analyzed by the logistic regression method. Significant variables and the classification performances of the models were determined. The performance in predicting disease outcome was evaluated by receiver operating characteristic (ROC) curve analysis. The optimum cut-off value was determined as the value with the highest sum of sensitivity and specificity values using the Youden index. *p* < 0.05 was considered statistically significant.

## 3. Results

This study encompassed a cohort of 106 patients, consisting of 49 females (46%) and 57 males (54%), with a mean age of 70.11 ± 14.23 years. The patient population was stratified based on prognosis, revealing that 95 individuals made up the survival group, while 11 patients experienced an unfavorable outcome, resulting in exitus. When the patients’ histories were examined, it was seen that 69 patients had hypertension, and 26 patients had congestive heart failure. Of these 26 patients, 9 had HFPEF (heart failure with preserved ejection fraction) and 17 had PHREF (heart failure with reduced ejection fraction). The demographic characteristics are summarized in Table 1.

To comprehensively evaluate the patients, the vital parameters, laboratory values, and blood gas results were meticulously analyzed. Strikingly, the sixth hour’s pH value and lactate clearance measurements exhibited statistically significant elevation in the survival group compared to the exitus group. However, the other parameters examined did not display significant variations based on the outcome status, as delineated in Table 2.

Of the patients in the survival group, 49.6% were successfully discharged following the completion of treatment and a subsequent follow-up, underscoring the clinical relevance of the survival outcome.

This study further probed the influence of clinical parameters on the outcome through logistic regression analysis. Remarkably, lactate clearance emerged as a pivotal factor significantly affecting outcome determination (χ^2^ = 19.827, *p* < 0.05). Model significance was affirmed by Cox–Snell R2 and Nagelkerke R2 exceeding 0.2. Additionally, the compatibility of the model with the data was confirmed with a *p*-value greater than 0.05 in the Hosmer–Lemeshow test. Notably, the coefficients associated with the initial lactate, sixth-hour PaO2, and BNP values were deemed nonsignificant, whereas the coefficients linked to lactate clearance held statistical significance. Each one-unit increase in lactate clearance was associated with a 5.55-fold increase in the probability of survival, as detailed in Table 3.

**Figure 2:**
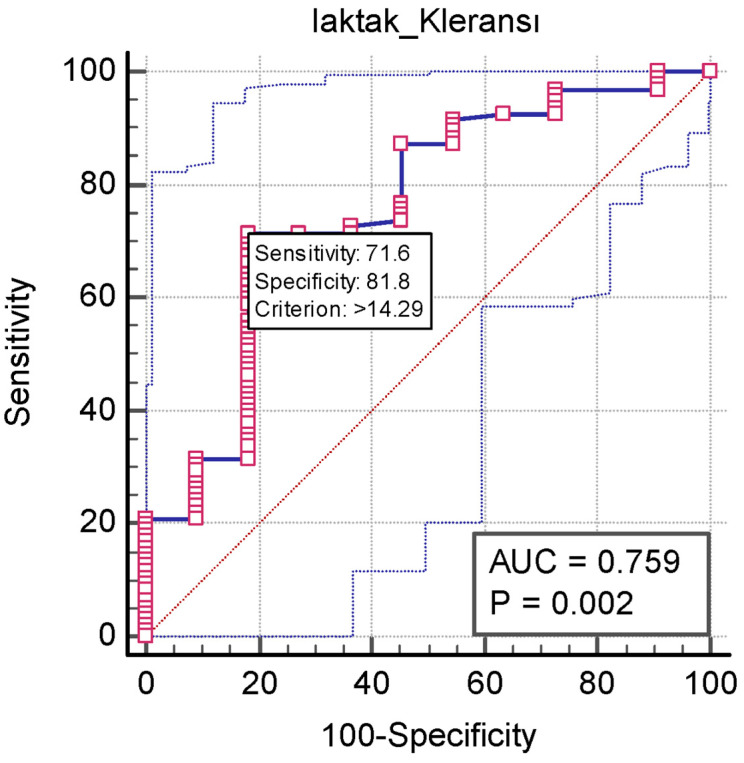
ROC curve for LC measurement performance in predicting outcome.

The discriminatory power of lactate clearance as a prognostic indicator was reflected in the calculated area under the curve (AUC) value of 0.759 (*p* < 0.001). The optimal cut-off value for lactate clearance was determined to be >14.29, demonstrating a sensitivity of 71.58% and a specificity of 81.82%, elucidated in Table 4. The corresponding ROC curve graphs for lactate clearance are visually represented in Figure 2.

**Figure 3:**
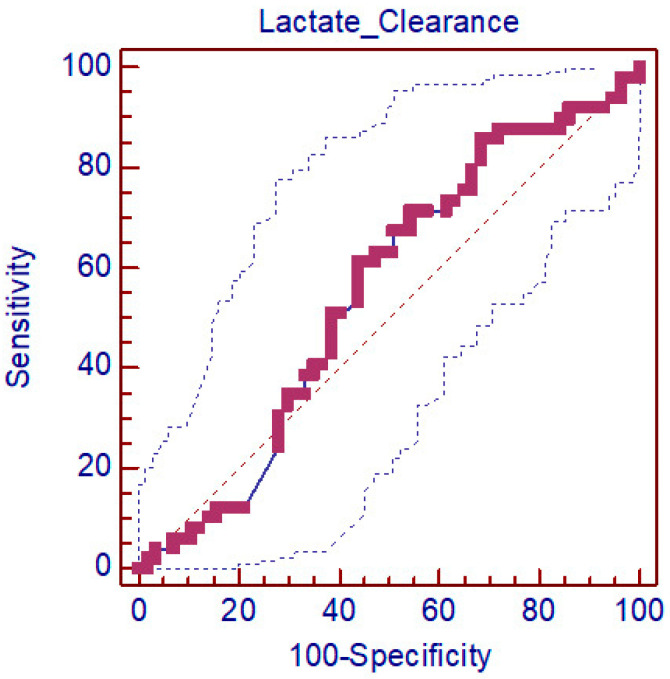
ROC analysis for LC success in predicting discharge.

However, caution is warranted when interpreting lactate clearance as a standalone prognostic marker for predicting discharge from the emergency department in patients with acute cardiogenic pulmonary edema, as indicated by the ROC analysis model. The predictive success of lactate clearance for discharge was not statistically significant, emphasizing the need for a holistic approach in prognostic assessment, as presented in Table 4 and Figure 3.

## 4. Discussion

The most important result of this study was that lactate clearance was a rapid, predictive, and applicable value in patients with decompensated heart failure due to cardiogenic pulmonary edema, with values below 14.29% indicating a poor prognosis and values above 14.29% indicating a good prognosis.

Although the underlying cause and pathophysiology vary in fatal diseases, the cause of lactate elevation is tissue hypoxia, vasodilatation, and the release of inflammatory mediators [13]. In the study by Nguyen HB et al. [13], an increased LC was found to be associated with a good outcome in patients with sepsis and septic shock. Nguyen HB et al. found that a change in the LC value of more than 10% after 6 h of treatment following current protocols had a sensitivity of 44.7% and a specificity of 84.4% in predicting in-hospital mortality. In a similar study, Marty et al. observed that a value of >20% significantly decreased the 28-day mortality rate in patients with septic shock [18].

Our findings revealed that the lactate clearance value is an important prognostic indicator in determining outcomes. Lactate is directly related to tissue hypoxia. Therefore, lactate elevation is an inevitable result of patients with cardiogenic pulmonary edema presenting to the emergency department, causing hypoxic respiratory failure. Marbach JA et al. investigated the usefulness of LC for mortality prediction in patients with cardiogenic shock and concluded that LC could be used to predict survival [19]. After appropriate treatment, lactate values regress with the improvement in hypoperfusion. LC, which allows us to measure the value of this regression, appears to be a sensitive, easily calculable, and reliable parameter for predicting outcomes in patients presenting with cardiogenic pulmonary edema.

On the other hand, when we consider the second outcome of our study, we can observe that, for discharging a patient from the emergency service, lactate clearance alone is not enough as a basis for this decision. Contrary to our study, Durmuş U et al. [20] observe that lactate clearance is a valuable marker in deciding whether to discharge or hospitalize patients with COPD exacerbation. From this example, we can say that lactate clearance changes in cardiogenic and pulmonary decompensation may have different outcomes, and the most important thing is to provide the patient at hand with a correct diagnosis.

In the study conducted by Chertoff J. et al. [21], it was found that measuring lactate clearance after 24–48 h was significant in the early recognition of sepsis and septic shock to predict mortality and the decision to initiate vasopressor drugs. This study determined again the importance of using lactate clearance for early diagnosis and rapid treatment initiation. We think that this may be a common result in all cases where the metabolic rate increases.

This study determined that the cut-off point for lactate clearance in patients admitted with pure cardiogenic pulmonary edema is 14.29%. In other studies, researchers observed varying thresholds for diagnosing different conditions. For example, the recommended threshold for 6 h lactate clearance in septic patients is 36%, notably higher than previously documented findings. Furthermore, this threshold significantly correlates with the mortality rate within 30 days, as demonstrated by independent studies [22]. Additionally, the measurement of the blood lactate levels at the onset and the subsequent assessment of lactate kinetics after 6 h have been identified as important indicators of the severity of shock. A threshold of 38.1% has been determined to be the optimal value for predicting survival in patients with shock, as reported in a study [23]. A study involving pediatric subjects discovered that a 24 h lactate clearance cut-off of 10% is a reliable indicator for predicting in-hospital mortality in children diagnosed with septic shock [24]. Consistent with the findings of our study, a separate investigation conducted on a cohort of critically ill children demonstrated that the measurement of lactate clearance at the 6 h mark is a dependable and accurate indicator for predicting mortality. Specifically, a threshold value of 16.435% was identified, beyond which a high likelihood of mortality was observed [25]. As indicated, the LC cut-off values for predicting mortality change in different illnesses and ages. Unfortunately, because of the variables (age, comorbidity, manner of presentation to the hospital) in this subject, our knowledge is not proper and precise.

This study has some limitations. The most important limitation is that the survey was single-centered. However, subgroups with more significant numbers should be formed for patients with chronic renal and chronic hepatic failure. Although our results are reliable, multicenter studies with more patients are needed.

## 5. Conclusions

In this study, lactate clearance below 14.29% was significantly associated with mortality in patients with acute cardiogenic pulmonary edema. We think that this value is important for starting treatment faster upon a patient’s first presentation to the emergency department. Studies with a higher number of patients will provide clearer values on this subject.

## Figures and Tables

**Table 1:** Comparison of demographic characteristics by outcome.

	Outcome		Test Statistics	
	Ex *n =* 11	Survivor *n =* 95	Test Value	*p*
**Age**, (*Years*)			−0.681 ^‡^	0.496
*Mean* ± *SD*	67.18 ± 16.04	70.43 ± 10.9		
*M* (*min–max*)	65 (38–93)	70 (43–98)		
**Gender**, *n (%)*			0.268 ^†^	0.605
Male	5 (45.5)	51 (53.7)		
Woman	6 (54.5)	44 (46.3)		
**Chronic disease**, *n (%)*			2454 ^†^	0.117
No	4 (36.4)	16 (16.8)		
Yes	7 (63.6)	79 (83.2)		
**Diabetes**, *n (%)*	4 (22.2)	33 (19.2)	7699 ^†^	0.174
Hypertension	5 (27.8)	64 (37.2)		
Coronary artery disease	2 (11.1)	40 (23.3)		
Congestive heart failure	6 (33.3)	20 (11.6)		
Chronic renal failure	1 (5.6)	10 (5.8)		
Malignancy	0 (0)	5 (2.9)		

^†^: Chi-square test; ^‡^: Mann–Whitney U Test (z). Summary statistics are given as a mean ± standard deviation and the median (minimum, maximum) for numerical data, and as a number (percentage) for categorical data.

**Table 2:** Comparison of clinical findings by outcome.

	Outcome		Test Statistics	
	Ex *n =* 11	Survivor *n =* 95	Test Value	*p*
**Hospitalization**			**12,173** ^†^	**0.002**
On foot	0 (0)	49 (51.6)		
Service	3 (27.3)	20 (21.1)		
Intensive care	8 (72.7)	26 (27.4)		
**Systolic blood pressure**			−0.700 ^‡^	0.484
*Mean* ± *SD*	170.45 ± 29.29	192.79 ± 149.58		
*M* (*min–max*)	157 (140–220)	188 (100–1600)		
**Diastolic blood pressure**			−1.303 ^‡^	0.193
*Mean* ± *SD*	82.82 ± 24	92.2 ± 20.7		
*M* (*min–max*)	89 (43–120)	94 (11–160)		
**Pulse**			−0.933 ^‡^	0.351
*Mean* ± *SD*	93.64 ± 27.43	97.86 ± 18.19		
*M* (*min–max*)	88 (60–140)	95 (60–160)		
**First measured pH**			−1.643 ^‡^	0.100
*Mean* ± *SD*	7.24 ± 0.12	7.29 ± 0.13		
*M* (*min–max*)	7.26 (6.99–7.41)	7.31 (6.97–7.54)		
**First measured satO2**			−1.643 ^‡^	0.100
*Mean* ± *SD*	91.05 ± 8.9	81.78 ± 19.41		
*M* (*min–max*)	94 (73–99.8)	89 (23–99)		
**First measured PaO2**			−1.177 ^‡^	0.239
*Mean* ± *SD*	130.05 ± 169.75	79.11 ± 48.53		
*M* (*min–max*)	60 (51.2–630)	61.5 (21–250)		
**First measured PaCO2**			−0.171 ^‡^	0.864
*Mean* ± *SD*	47.78 ± 15.89	46.49 ± 13.08		
*M* (*min–max*)	45 (33–87)	46 (8.5–89)		
**First measured Lactate**			−1.933 ^‡^	0.053
*Mean* ± *SD*	2.25 ± 2.76	3.03 ± 2.58		
*M* (*min–max*)	1.1 (0.6–9.6)	2.2 (0.4–15)		
**Sixth hour pH**			**−3.022** ^‡^	**0.003**
*Mean* ± *SD*	7.33 ± 0.06	7.4 ± 0.07		
*M* (*min–max*)	7.31 (7.27–7.44)	7.4 (7.12–7.55)		
**Sixth hour Sat O2**			−1.617 ^‡^	0.106
*Mean* ± *SD*	93.97 ± 4.35	87.96 ± 12.96		
*M* (*min–max*)	93.3 (86.3–99.1)	92 (30–99.8)		
**Sixth hour PaO2**			**−1.989** ^‡^	**0.047**
*Mean* ± *SD*	139.83 ± 129.59	72.64 ± 34.15		
*M* (*min–max*)	70.2 (51–394)	65 (24–218)		
**Sixth hour PaCO2**			−1.119 ^‡^	0.263
*Mean* ± *SD*	43.6 ± 11.77	39.1 ± 7.64		
*M* (*min–max*)	41.6 (28.6–65)	39 (24.8–65)		
**Sixth hour Lactate**			0.001 ^‡^	0.999
*Mean* ± *SD*	2.36 ± 2.49	1.51 ± 0.88		
*M* (*min–max*)	1.5 (0.6–8.3)	1.3 (0.4–5.5)		
**BNP**			−1.746 ^‡^	0.081
*Mean* ± *SD*	636.11 ± 190.09	891.2 ± 669.74		
*M* (*min–max*)	601 (422.27–968)	731 (73.85–4700)		
**Lactate clearance**			**−2804** ^‡^	**0.005**
*Mean* ± *SD*	−28.69 ± 79.26	28.08 ± 46.31		
*M* (*min–max*)	−5.77 (−216.67–66.67)	34.09 (−175–88.89)		

^†^: Chi-square test; ^‡^: Mann–Whitney U Test (z). Summary statistics are given as a mean ± standard deviation and the median (minimum, maximum) for numerical data, and as a number (percentage) for categorical data. The results highlighted in bold are statistically significant (*p* < 0.05).

**Table 3:** Examination of effective findings on outcome logistic regression (χ^2^). β: regression coefficient; se: standard error; zβ: odds ratio (OR); and R^2^: coefficient of determination. The bold sections are statistically significant (*p* < 0.05).

	Regression Coefficients *						
	*β*	*if*	*zβ*	*wald*	*p*	95.0% Confidence Interval for *β*	
						*Lower Bound*	*Upper Bound*
**Model: Outcome (0-Ex; 1 = Survivor)**							
**constant**	0.789	1248	0.400	2,202	0.527		
**Lactate 0**	−0.061	0.162	0.143	0.940	0.705	0.68	1.29
**PaO2 6**	−0.009	0.005	3106	0.991	0.078	0.98	1.00
**BNP**	0.003	0.002	3652	1003	0.056	1.00	1.01
**Lactate Clearance**	0.019	0.008	**5555**	1019	**0.018**	1.00	1.04

**Model Summary:** −2 Log likelihood = 50,832; Cox & Snell R^2^ = 0.171; Nagelkerke R^2^ = 0.351; χ^2^ = 19.827; *p* = 0.001. * **Hosmer and Lemeshow Test:** χ^2^ = 4,665; *p* = 0.793.

**Table 4:** A-ROC analysis Lactate clearance measurements’ performance in predicting outcome and B-ROC analysis for LC success in predicting discharge.

	***AUC*** **(95% CI)**		***p***		**Youden Index J**	**Cut-Off**	**Sensitivity (95% CI)**	**Specificity (95% CI)**	
Lactate Clearance	0.759 (0.666–0.837)		0.002		0.534	>14.29	71.58 (61.4–80.4)	81.82 (48.2–97.7)	
	**Area under the Curve (*AUC*)**	***if***		***p***	**Area under the Curve (*AUC*) 95% Confidence Limits**		**Sensitivity**	**Selectivity**	**Limits**
					**Lower Limit**	**Upper Limit**			
Lactate Clearance	0.550	0.056		0.375	0.451	0.647	61.22	56.14	≤31.58

*AUC*: area under the curve; CI: confidence interval.

## Data Availability

The original contributions presented in the study are included in the article, further inquiries can be directed to the corresponding author/s.
